# General Versus Regional Anaesthesia for Lower Limb Arthroplasty and Associated Patient Satisfaction Levels: A Prospective Service Evaluation in the Oxford University Hospitals

**DOI:** 10.7759/cureus.17024

**Published:** 2021-08-09

**Authors:** Gregory Neal-Smith, Erin Hopley, Lysander Gourbault, Daniel T Watts, Harry Abrahams, Katy Wilson, Vassilis Athanassoglou

**Affiliations:** 1 Orthopaedics, Oxford University Hospitals NHS Foundation Trust, Oxford, GBR; 2 Anaesthetics, Oxford University Hospitals NHS Foundation Trust, Oxford, GBR

**Keywords:** total joint arthroplasty, general anaesthesia, regional anaesthesia, patient satisfaction, arthroplasty lower limb, service evaluation

## Abstract

Introduction

Lower limb arthroplasty is performed under general anaesthesia (GA) or regional anaesthesia (RA). There is increasing evidence of the surgical and anaesthetic benefits of RA. National Institute for Health and Care Excellence (NICE) guidelines advise using either but highlight a lack of data comparing outcomes of RA and GA for these procedures. We conducted a service evaluation, prospectively analysing elective orthopaedic cases performed at the Nuffield Orthopaedic Centre, Oxford, UK from 19/11/2018 to 03/04/2019. We aimed to compare data on anaesthetic assessment, intra-operative parameters and patient satisfaction for RA and GA cases.

Methods

We selected elective patients, aged above 18, undergoing total hip, total knee or unilateral knee arthroplasties. Prospective quantitative and qualitative data were collected using two forms. Firstly, anaesthetists completed a case report recording demographic data, intra-operative details and reason for anaesthetic choice. Secondly a questionnaire gathered patient satisfaction data. This was analysed using descriptive statistics and presented in tables.

Results

Data for 132 patients were collected over the service evaluation period. After exclusion, 99 patients were included for final analysis; 59 underwent GA and 40 had RA. GA was used predominantly due to patient preference (74.6%). RA was used primarily due to anaesthetic preference (75%); most commonly due to speed of list and duration of operation. Overall patients had low pain scores (0.3/10) and high pre-operative anxiety levels (4.6/10) regardless of anaesthetic.

Conclusion

Our results show high patient satisfaction with GA and RA for lower limb arthroplasty; however, pre-operative anxiety was common for both. Patient preference and comfort influenced choice of anaesthesia, highlighting the importance of pre-operative counselling and education to facilitate shared decision making, leading to favourable post-operative outcomes.

## Introduction

Total joint arthroplasty is one of the most commonly performed procedures in developed countries [1]. The National Health Service performs over 145,000 hip and knee arthroplasty procedures per year in the UK [2] and this number is predicted to increase further [3]. Lower limb arthroplasty can be performed under general anaesthesia (GA) or with regional anaesthesia (RA). RA is used to describe the targeted administration of an anaesthetic agent to numb specific parts of the body; this term encompasses central neuraxial blocks (CNB) and peripheral nerve blocks (PNB). Recent evidence and international consensus recommend the use of RA in lower limb arthroplasty due to several postoperative outcome benefits [1]. For the anaesthetist, cardiovascular and respiratory stability, as well as rapid postoperative recovery are important advantages of RA [2,3]. In terms of surgical outcomes, many studies have shown that RA provides superior perioperative analgesia, reduces post-operative nausea and vomiting, enhances patient recovery & often results in increased patient satisfaction. For these reasons, RA (with or without sedation) has become an attractive alternative to general anaesthesia (GA), and is increasingly used for orthopaedic surgery. These outcomes have been reviewed in detail by Hutton [4] and Kubulus et al [5].

To date, there is limited data on the frequency at which GA and RA are used for elective orthopaedic surgery. The most recent comprehensive analysis, published in 2015, presented data pulled from key orthopaedic surgery data banks including the National Hip Fracture Database (NHFD) and Global Orthopaedic Registry (GLORY) [6]. Overall, the UK used more RA compared to the United States of America (USA) and some European countries, with a two-fold increase in the utilisation of peripheral nerve blocks over the last two decades in both inpatient and ambulatory orthopaedic surgery. Despite this, however, all studies showed that > 50% of orthopaedic procedures in the UK are still done under GA.

Updated National Institute for Health and Care Excellence (NICE) guidelines on anaesthesia for primary joint replacements (hip, knee and shoulder) were released in June 2020 [7]. The guidelines advise for both hip and knee arthroplasty using regional anaesthesia in combination with local infiltration analgesia (LIA) or GA with an LIA [7]. As part of this, they highlight the lack of large data sets, and prioritise the need to look at clinical outcomes and cost-effectiveness of nerve blocks, periarticular infiltration and GA compared with each other, alone and in combination [8]. This shift in scope is in keeping with the updated recommendations from the International Consensus on Anaesthesia-Related Outcomes After Surgery Group (ICAROS) - they recommend regional anaesthesia for knee and hip arthroplasty (evidence level: moderate-low) and emphasise the need for further studies to strengthen future meta-analyses [3].

Due to the identified lack of data, we conducted a service evaluation, where we analysed elective orthopaedic cases performed at the Nuffield Orthopaedic Centre, Oxford, UK, from 19/11/2018 to 03/04/2019. For each case, we collected data on: Anaesthetic assessment including patient demographics and clinical reasoning for selected anaesthetic technique. Also overall patient satisfaction including feedback on the operation and anaesthetic technique used. In order to conduct a project with prospective data collection at this scale, we recruited help from local medical students.

## Materials and methods

This prospective service evaluation took place between 19/11/2018 and 03/04/2019 at the Nuffield Orthopaedic Centre, Oxford, United Kingdom. We selected procedures which were amenable to either general anaesthesia or regional anaesthesia. These included consecutive cases of total hip replacements (THR), total knee arthroplasties (TKA) and unilateral knee arthroplasties (UKA) in patients aged 18 years and above. Ethical approval for this service evaluation was obtained from Oxford University Hospitals Research & Development Team.

Anaesthetists were asked to complete a case report form which included patient demographic data including age, gender, body mass index (BMI, kg/m^2^), and American Society of Anesthesiologists (ASA) physical status classification. In addition, this assessed primary anaesthetic technique and justification for chosen modality (Appendix A). Following their operation, these same patients were then asked to complete a patient satisfaction questionnaire designed specifically for this project whilst in recovery, which was typically within hours of their procedure. This evaluated their anxiety levels before, during and after the operation with interval rating scales and compared their current anaesthetic experience with previous occasions where applicable (Appendix B). This facilitated collection of quantitative and qualitative data.

Thirty-five Oxford University medical students were recruited as collaborators. This aided with patient identification and engagement with staff to maximise form completion. The case report form and patient satisfaction questionnaire data were assessed using basic descriptive statistics with representative qualitative data presented in tables.

## Results

Data for 132 patients were collected over the service evaluation period. Thirty-three patients were excluded due to insufficient information on the clinician reported outcome form, namely lack of operation name or type of anaesthesia. In addition, 54 of these patients also completed a patient satisfaction questionnaire. Patient demographic details, operation and anaesthetic type can be found in Table 1.

**Table 1:** Demographic data of patients. Values are means/totals (ranges). ASA: American Society of Anesthesiologists; GA: general anaesthetic; RA: regional anaesthesia; Sedation - examples include benzodiazepines and opioids.

	Total (99)	GA (59)	RA & sedation (35)	RA alone (5)
Gender (F), n (%)	53 (53.5)	36 (61)	15 (42.9)	2 (40)
Age mean (range)	66 (27-86)	64.3 (27-86)	69.7 (49-84)	64.2 (44-76)
BMI mean (range)	29.2 (21-50.8)	29.5 (21-43.4)	28.8 (22.7-37.4)	29 (22.2-50.8)
ASA mean (range)	2 (1-3)	2.1 (1-3)	2 (1-3)	2.5 (2-3)
Respiratory disease, n (%)	22 (22.2)	15 (25.4)	6 (17.1)	1 (20)

Reasons for anaesthetic choice

Patient preference was a frequent factor in choosing to perform a general anaesthetic over regional anaesthesia, with the use of an additional nerve block felt to help improve patient comfort (Table 2). When choosing to perform sedation with RA, anaesthetic and patient preference predominated as reasons given for anaesthetic choice. Surgeon preference rarely influenced anaesthetic choice however it was cited more frequently in cases where both GA and nerve block were utilised (15%). In addition, the duration of the operation alongside the speed of the list and anaesthesia were more highly cited as reasons to perform sedation plus RA. Using sedation and RA were part of the enhanced recovery pathway for TKA and THR.

**Table 2:** Reasons documented by anaesthetists for anaesthetic technique. Multiple reasons could be given per patient. GA: general anaesthetic; RA: regional anaesthesia; PNB: peripheral nerve block.

Anaesthetic technique (n)	Patient preference	Anaesthetic preference	Surgeon preference	Patient comfort	Comorbidities	Speed of anaesthesia and list	Duration of operation	Enhanced recovery	Other
GA only (26)	17	10	0	1	5	1	0	0	2
GA & PNB (33)	27	19	5	11	4	7	3	0	0
RA & sedation (35)	13	28	2	5	8	8	9	6	0
RA (5)	3	2	0	0	2	0	0	0	0

Patient experience

Patients were overall very comfortable regardless of anaesthetic modality, scoring themselves less than 1/10 in all groups for their pain scores (Table 3). Anxiety levels were high in all groups prior to the operation (4.6/5) which as one patient stated was “purely anxiety related to rational assessments of risks of surgery”. These anxiety levels significantly reduced in both the GA and RA & sedation groups both intraoperatively and postoperatively. This was not the case in the RA group where anxiety levels stayed high throughout. Patients in GA and RA & sedation groups were highly likely to recommend their received method of anaesthesia, 4.3/5 and 4.7/5, respectively. This was less commonly the case in the RA only group, 3.3/5 (Table 3).

**Table 3:** Patient average discomfort scores and recommendation of their received anaesthesia. Values are means (ranges). GA: general anaesthetic; RA: regional anaesthesia.

	GA (32)	RA & sedation (19)	RA (3)	All (54)
Discomfort score (/10)	0.4 (0-8)	0.4 (0-6)	1 (0-2)	0.3 (0-1)
Anxiety level before (/10)	5 (0-10)	3.7 (0-10)	6.3 (5-8)	4.6 (0-10)
Anxiety level during (/10)	0.8 (0-9)	1.3 (0-10)	6 (5-7)	1.2 (0-10)
Anxiety level after (/10)	2.5 (0-9)	1.8 (0-10)	6.5 (7-7)	2.4 (0-10)
Would recommend this method of anaesthesia (/5)	4.3 (1-5)	4.7 (3-5)	3.3 (2-5)	4.4 (1-5)

Patients in the regional anaesthesia group were grateful to have increased interaction and to avoid general anaesthetic side effects. However, they felt that they would have liked more information about what the procedure would entail and one patient was distressed at not being able to feel their legs. Patients receiving a general anaesthetic were overall very positive of their experience (Table 4). It was noticeable that these patients made significantly more comments relating to symptoms of nausea, fatigue and pain compared to the sedation group. A summary of their comments can be found in Table 4.

**Table 4:** Summary of patient’s experience of anaesthesia and suggested improvements GA: general anaesthetic; RA: regional anaesthesia.

	GA (32)	RA & sedation (19)	RA (3)
Experience of the operation	“I don’t remember” “excellent” "have felt nauseous"	“Very relaxed and good”; “No recollection of operation”; “Excellent”	"I slightly panicked about not being able to move, not explained that this would happen"; "Block very cold"
Experience compared to previous GA	“Better this time”; “Better, no nausea so far, less drowsy”; "No difference"	“Better”; “Spinal sedation this time was much better”; “Scary not to be able to feel my legs”	"Felt more involved and waking up afterwards was better"; "good"
What could have been done to improve your comfort	“Nothing"; "Pain was severe on waking"	“Nothing to improve”	"Not possible"
Could anything have been done to improve your anxiety?	“Nothing! Perfect Care!”; “Not having to wait helped”; “Waiting”; “Naturally anxious”	"Didn't quite know what to expect"; "I have been pleased with everything that's happened"	"Discussion about process/possibility of spinal anaesthesia and sedation at preop assessment to feel better prepared"

## Discussion

Patient satisfaction

This service evaluation demonstrates that both general and regional anaesthesia modalities led to high satisfaction levels in patients undergoing lower limb arthroplasty procedures. Currently, data on patient satisfaction related to anaesthetic choice perioperatively in lower limb arthroplasty is limited [1]. Moreover, there are few studies that have collected data prospectively [1]. Patient satisfaction is an important outcome measurement, as it influences healthcare delivery at a societal and individual level [9], and is considered an integral part of service quality [10]. Black et al have shown that better patient experience in elective surgery is associated with lower patient-reported complications relating to wounds, the urinary system, bleeding and drug reactions [11]. Improving patient outcomes can increase patient experience ratings by up to 10%, similarly improving patient experience ratings can improve outcome scores by up to 3% [12]. Our service evaluation adds prospectively collected data from a high-volume orthopaedic centre to help guide choices for patients and clinicians in the future.

The findings demonstrate that for general anaesthetic and non-general anaesthetic techniques (neuraxial anaesthesia), patients had high levels of satisfaction. This included different combinations of adjuncts including peripheral nerve blocks and sedation, with most patient-reported outcomes demonstrating equivalence. Similar findings have been noted in previous literature [13,14], with a randomised controlled trial by Harsten finding no difference in the anaesthetic satisfaction score between general and neuraxial anaesthetic groups [13]. However, significantly fewer patients in the general anaesthetic group would opt to change their method of anaesthesia for a subsequent operation compared with the neuraxial anaesthesia group [13].

Choice of anaesthesia

A systematic review by Johnson et al. called for studies focusing on surgeon and anaesthetist preferences in choosing neuraxial anaesthesia [1]. Our service evaluation has highlighted reasons for selection of neuraxial anaesthesia along with different anaesthetic modalities. A notable finding was that duration of operation alongside speed of the list was cited more frequently for reasons to perform neuraxial anaesthesia. Although many clinicians in our unit felt this led to increased speeds, the evidence in the literature is inconclusive. Johnson et al. found there is evidence neuraxial anaesthesia may be responsible for shorter surgical duration (up to 11 minutes) but this difference failed to achieve statistical significance (P = 0.08) [1]. These considerations are also relevant during the COVID-19 era, as tracheal intubation is considered an aerosol-generating procedure [14]. These procedures expose clinicians to the upper respiratory tract with high viral loads, which has been shown to increase the transmission of coronaviruses [14]. To reduce this risk, clinicians may consider performing neuraxial anaesthesia in selected patients, as an alternative to intubation in general anaesthesia.

Patient comfort was a highly cited reason for patients receiving peripheral nerve block and sedation. A recent Cochrane review found peripheral nerve blocks increase patient satisfaction, reduce pain on arrival in postoperative care units, and reduce hospital length of stay, compared with systemic analgesia. However, when compared with neuraxial anaesthesia, there was no difference [15]. Our results are concordant with Adhikary et al. who found that peripheral nerve blocks were used predominantly with general anaesthesia rather than neuraxial anaesthesia [16]. This may be linked to factors including requirement of systemic analgesia and ability to mobilise but this was not measured in this service evaluation. Furthermore, our service evaluation highlighted that surgeon preference rarely influenced anaesthetic choice, which matches findings from previous literature [17].

Other findings

Our data indicate that for all procedures and anaesthetic modalities, anxiety was very high preoperatively. Given anxiety had substantially lowered after the procedure for all operations, despite many risks still being present, it suggests that there could be areas of improvement in preoperative counselling. Increased anxiety preoperatively is associated with detrimental pathophysiological responses such as hypertension, dysrhythmias and increased need for postoperative analgesia [18]. It has been shown that providing patients with video-based and printed information regarding anaesthesia preoperatively reduces anxiety levels [18]. A lack of patient education could also explain why more patients opted for general anaesthesia over regional anaesthesia. This service evaluation highlights the importance of comprehensive patient education and we would advocate provision of supplementary material to aid in this process.

We would also highlight the benefits of using a student cohort to help collect data. Research has found that medical students struggle to engage in research and audit work [19]. Collaborative approaches increase engagement and allow a large amount of clinical data to be collected prospectively which can be challenging with smaller teams. Similar success has been noted with student-led national collaboratives, including the Outcomes After Kidney Injury in Surgery (OAKS) and STARSURG1 publications led by STARSurgUK [19,20]. Involvement in research projects like these has been shown to improve academic performance, develop understanding of research and data collection methods which many will utilise as a junior doctor [19]. Quality improvement projects are also strongly encouraged by the GMC and this helps obtain these competencies.

Limitations

Due to clinical commitments, it was difficult capturing all patient data in the study period, which may have skewed results. Thirty-three patients (25%) were excluded due to insufficient information on the clinician case report forms. Secondly, patient satisfaction questionnaires were only fully completed by 54 (55%) of the 99 patients, due to a combination of patient refusal and inability to complete given recent anaesthetic, which highlights a significant loss to follow up. As a result, comparative data analysis was not performed due to a lack of patient numbers. Recall bias was another limitation, however it was aimed to minimise this as much as possible with data collection during hospital stay. In addition, neuraxial anaesthesia has been shown to significantly reduce the length of hospital stay, which could improve patient satisfaction [1]. This could have been reflected by collecting additional data after discharge.

Future development

We would encourage future studies to include multiple sites with larger sample sizes to allow for measurement of statistical significance on patient satisfaction in relation to anaesthetic modalities. Topics to explore in future research could include targeting anxiety levels preoperatively to improve patient experience, as mean anxiety scores in this patient cohort were very high prior to their operation. In addition, research regarding why patients are satisfied with different anaesthetic methods in lower limb arthroplasty could benefit clinical decision-making. 

## Conclusions

This service evaluation has prospectively collected data from a high-volume orthopaedic centre and shown that high satisfaction levels are associated with both general and regional anaesthesia in lower limb arthroplasty. The reason for selecting particular anaesthetic modalities was influenced significantly by patient preference, patient comfort and list efficiency. Preoperative anxiety levels were very high and the importance of preoperative education and counselling is highlighted. This could be targeted with provision of educational materials to aid patient decision-making and improve perioperative pathophysiology. Student cohorts have demonstrated they are an effective method for prospectively collecting large amounts of patient-reported qualitative data over an extended period of time. Future studies should target pre-operative anxiety levels and collect data across multiple sites to provide more information on patient experience in different anaesthetic modalities.

## Figures and Tables

**Figure 1:**
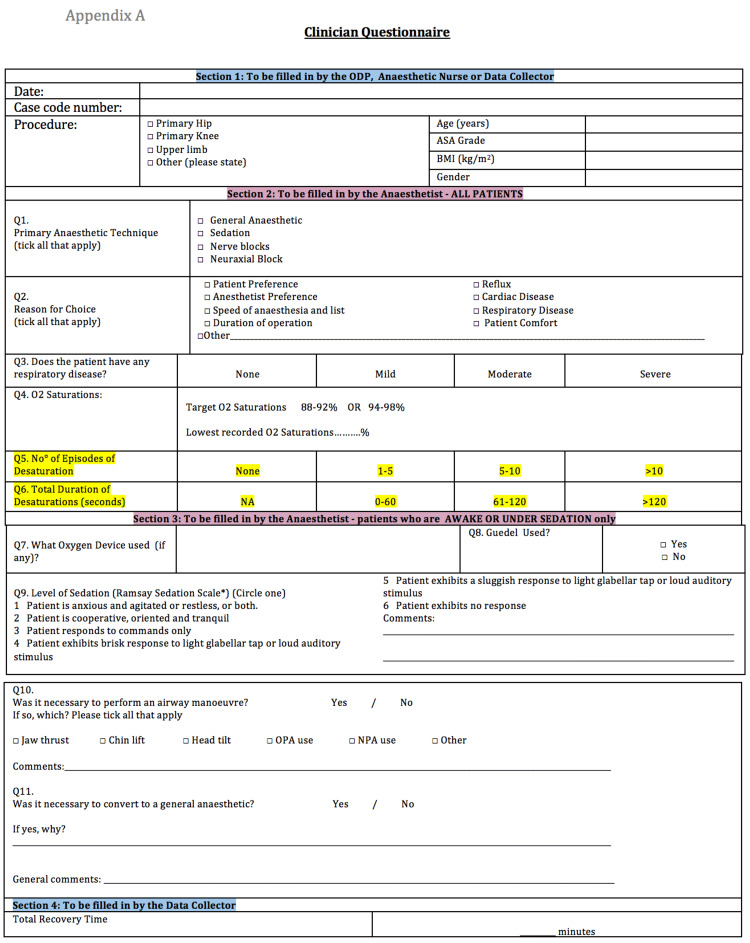
Clinician questionnaire.

**Figure 2:**
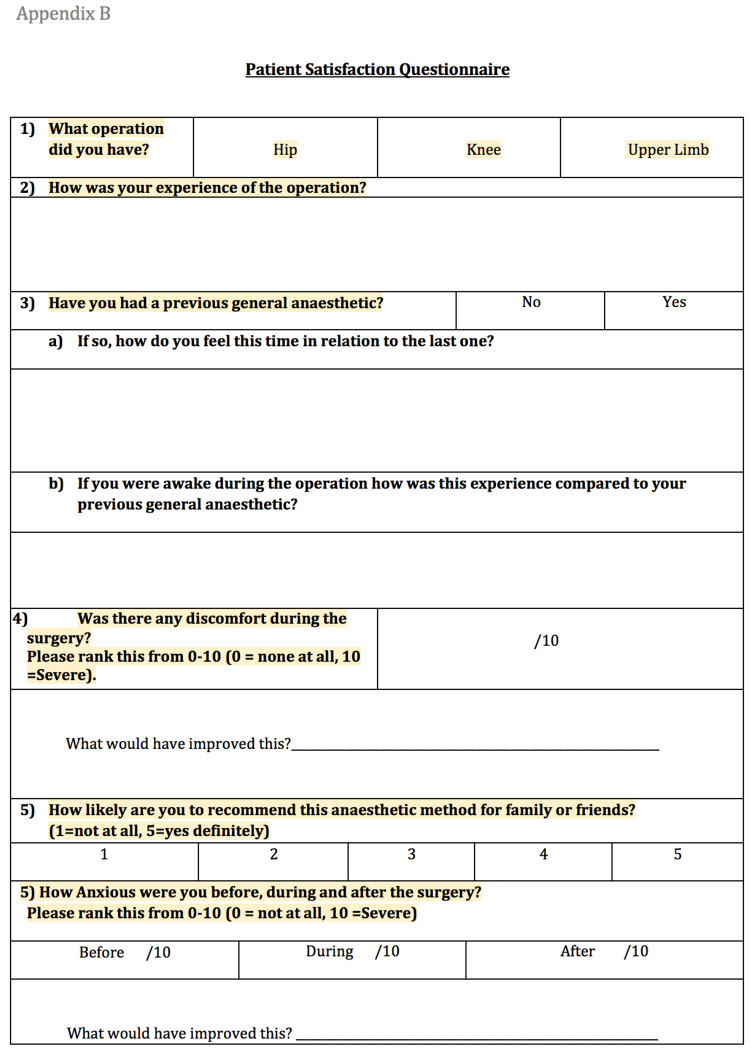
Patient satisfaction questionnaire.
